# Mitochondrial Dysfunction in Oxidative Stress‐Mediated Intervertebral Disc Degeneration

**DOI:** 10.1111/os.13302

**Published:** 2022-06-08

**Authors:** Dian‐Kai Wang, Huo‐Liang Zheng, Wen‐Sheng Zhou, Zheng‐Wei Duan, Sheng‐Dan Jiang, Bo Li, Xin‐Feng Zheng, Lei‐Sheng Jiang

**Affiliations:** ^1^ Department of Spine Centre Xinhua Hospital Affiliated to Shanghai Jiao Tong University School of Medicine Shanghai China; ^2^ Department of Orthopedics Xinhua Hospital Affiliated to Shanghai Jiao Tong University School of Medicine Shanghai China

**Keywords:** Intervertebral disc degeneration, Mitochondrial dynamics, Mitochondrial dysfunction, Mitophagy, Oxidative stress, Reactive oxygen species

## Abstract

Intervertebral disc degeneration (IVDD) is the most common contributor to low back pain (LBP). Recent studies have found that oxidative stress and reactive oxygen species (ROS) play an important role in IVDD. As a by‐product of aerobic respiration, ROS is mainly produced in the mitochondria by the electron transport chain and other mitochondrial located proteins. With the excessive accumulation of ROS, mitochondria are also the primary target of ROS attack in disc cells. A disrupted balance between intracellular ROS production and antioxidant capacity will lead to oxidative stress, which is the key contributor to cell apoptosis, cell senescence, excessive autophagy, and mitochondrial dysfunction. As the pivotal ingredient of oxidative stress, mitochondrial dysfunction manifests as imbalanced mitochondrial dynamics and dysregulated mitophagy. Mitochondria can alter their own dynamics through the process of fusion and fission, so that disabled mitochondria can be separated from the mitochondrial pool. Moreover, mitophagy participates by clearing these dysfunctional mitochondria. Abnormality in any of these processes either increases the production or decreases the clearance of ROS, leading to a vicious cycle that results in the death of intervertebral disc cells in large quantities, combined with degradation of the extracellular matrix and overproduction of matrix metalloproteinase. In this review, we explain the changes in mitochondrial morphology and function during oxidative stress‐mediated IVDD and highlight the important role of mitochondria in this process. Eventually, we summarize the IVDD therapeutic strategies targeting mitochondrial dysfunction based on current understanding of the role of oxidative stress in IVDD.

## Graphical abstract

Graphic overview of the role of mitochondrial dysfunction in the pathogenesis of IVDD. An imbalance between ROS intercellular production and antioxidant defense capacity will directly give rise to the occurrence of oxidative stress, which is mainly represented by cell apoptosis, abnormal autophagy, cell senescence, and mitochondrial dysfunction. Among the oxidative stress‐related pathophysiological processes, mitochondrial dysfunction plays an essential role in the disc pathology of IVDD, which manifests as imbalanced dynamics and dysregulated mitophagy. ROS promotes the fusion of mitochondria, which decreases their fission process, so that disabled mitochondria can be separated from the mitochondrial pool. Further, mitophagy serves as the quality control mechanism, which can be dysregulated under the condition of oxidative stress by overwhelmingly clearing both dysfunctional and normal mitochondria. Overall, the effects of mitochondria dysfunction result in the massive disc cell death with overproduction of MMP and the degradation of ECM, eventually the pathogenesis of IVDD.

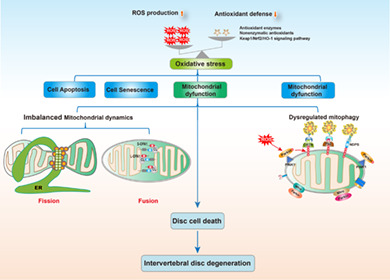

## Introduction

Intervertebral disc degeneration (IVDD) is characterized by an imbalance between extracellular matrix (ECM) synthesis and degradation as well as increased apoptosis and senescence of nucleus pulposus (NP) cells.^1^, ^2^, ^3^ The etiology of IVDD is very complex, with multiple factors such as aging, smoking, infection, excessive stress, diabetes, and trauma contributing to its pathogenesis.^4^, ^5^ IVDD has been widely accepted as a contributor to low back pain (LBP), the latter is a very common disorder that occurs in adults and is associated with a massive socioeconomic burden.^6^, ^7^

The intervertebral disc is the largest avascular structure in the human body,^8^ with some NP cells be located 6–8 mm from the nearest blood supply.^9^, ^10^ NP cells produce proteoglycan and type II collagen and control ECM metabolism, making them the most important functional cells in the intervertebral disc.^11^ Due to the lack of vascular structure, nutrients and metabolites are driven by diffusion gradients of glucose, oxygen, lactic acid, and other large molecules.^12^ Oxygen concentration gradients were found to drop by more than 90% from the anulus edge towards the disc center.^13^ Because of this hypoxic microenvironment, it has been thought that oxidative reactions do not occur in NP cells and that disc degeneration is not correlated with oxidative stress. However, recent studies have found that oxidative stress and reactive oxygen species (ROS) play an important role in IVDD.^14^

Mitochondria are an essential source of ATP, in addition to being the main ROS source in cells.^15^ When mitochondria are damaged, excessive stress signals are produced that lead to cell dysfunction and eventually programmed cell death.^16^, ^17^ Little attention has been paid to the role of mitochondrial dysfunction in this pathological process. This review provides an overview of the morphological and functional changes underlying mitochondrial dysfunction during oxidative stress in the pathogenesis of IVDD, and describes its significance for IVDD therapy.

## Methods

### Description of Searching Method

**Fig. 1:**
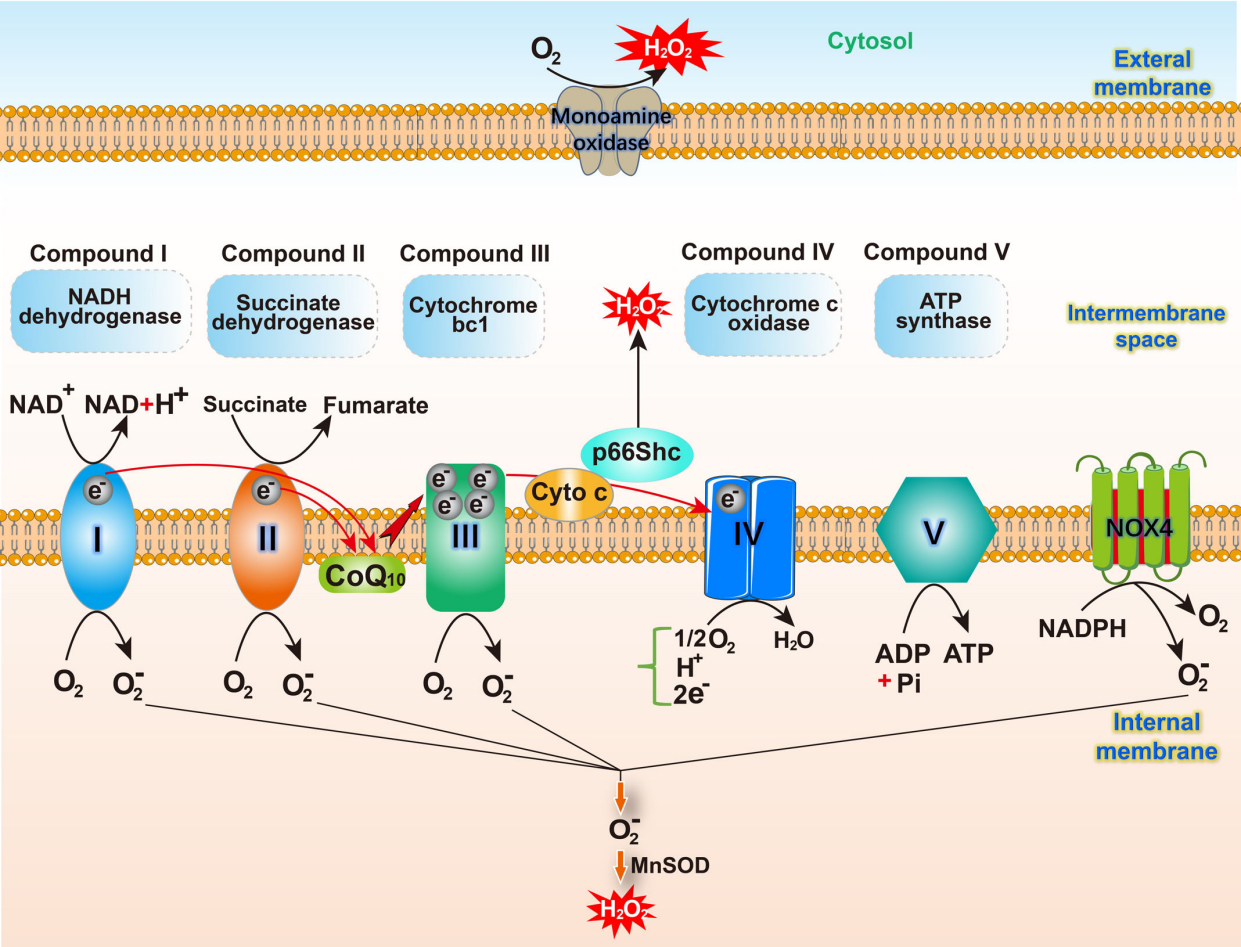
Flow chart of literature selecting process.

- Searching platforms: web of science, Pubmed, Google scholar;
- Databases: Embase, Medline;
- Key words: intervertebral disc degeneration, reactive oxygen species (ROS), ROS in disc cell(s), oxidative stress, mitochondrial dysfunction, mitophagy;
- Retrieving time: 2010–2019; and
- Excluded based on abstracts: cadaveric studies, *in‐vitro* studies, non‐English abstracts (Figure 1).

## Relationships between ROS and IVDD

ROS is a family of unstable and highly reactive molecules with or without free radicals including superoxide anions (O_2_^−^), hydrogen peroxide (H_2_O_2_), hydroxyl radicals, (OH^−^), hypochlorite ions (OCl^−^), nitric oxide (NO), and singlet oxygen. ROS generation is up‐regulated by external stimuli including pro‐inflammatory cytokines, nutrition deprivation, and mechanical loading.^14^ Disc cells are thought to be anaerobic because of the lack of blood supply to disc tissue, but virtually all disc cells (NP, annulus fibrosus [AF], and cartilage endplate [CEP] cells) have been shown to metabolize oxygen and use oxidative phosphorylation for energy production *in vivo*.^18^, ^19^, ^20^ Moreover, ROS and peroxisomes have been identified in human NP cells.^21^, ^22^ Recent research has also confirmed that an imbalance between excessive ROS generation and inherent antioxidant capacity leads to IVDD.^14^

Apoptosis is a form of programmed cell death implicated in the large reduction in NP cells number under pathophysiological conditions.^23^ There are two main caspase‐dependent signaling pathways leading to NP cell apoptosis: the mitochondrial and death‐receptor pathways.^24^ Studies have confirmed that oxidative stress is an important factor for the induction of NP and AF cells apoptosis through the mitochondrial pathway.^25^, ^26^ Elevated intracellular ROS levels can inhibit the expression of B‐cell lymphoma‐2 (Bcl‐2), leading to changes in mitochondrial membrane permeability and release of apoptosis‐related signaling molecules, such as cytochrome c. Cytochrome c initiates formation of the apoptosome—consisting of cytochrome c, caspase‐9, and apoptotic protease‐activating component (Apaf‐1)—which activates caspase‐3 and triggers NP and AF cells apoptosis.^27^, ^28^

Autophagy is an evolutionarily conserved lysosomal activity characterized by the degradation of damaged intracellular organelles and metabolites, while providing energy for cells at the same time.^29^ Autophagy is the basis for cell survival, but excessive autophagy leads to excessive lysosomal degradation of cell constituents, eventually resulting in cell death. ROS can induce autophagy through the ERK/m‐TOR signaling pathway in NP cells.^26^ In rat notochordal cells, ROS was found to increase Beclin‐1, LC3‐II, and Atg3, 5, 7, and 12 levels, leading to autophagy.^30^ The autophagy of notochordal cells is recognized as the starting point of IVDD.^14^

Cell senescence is a process of irreversible cell cycle arrest that leads to decreased activity of disc cells. The accumulation of senescent disc cells is another trigger of IVDD.^31^ Senescent disc cells promote the secretion of matrix proteases, inflammatory factors, and chemokines, defined as senescence‐associated secretory phenotype (SAAP), worsening the disc microenvironment. The presence of H_2_O_2_ in NP cells has also been demonstrated, the concentration of which is dose‐dependent upon the degree of cell senescence. Upon H_2_O_2_ accumulation, the expression of two classical senescence markers, p21WAF1 and p16INK4a, is increased in NP cells.^32^ H_2_O_2_ also leads to senescence of human CEP cells through the p53‐p21‐Rb pathway.^33^ In addition to decreased cell function, cell senescence increases extracellular matrix decomposition. *In vitro* and organotypic studies have shown that ROS leads to an increase in catabolic markers such as ADAMTS‐5 and the matrix metalloproteases MMP‐1 and MMP‐3.^34^ ROS disturb the balance between matrix anabolism and catabolism, and remarkedly reduce the matrix content of discs.

ROS plays an important role in the pathological process of IVDD by mediating excessive autophagy, apoptosis, and senescence of disc cells, especially in NP cells, through various signaling pathways such as the MAPK, ERK/m‐TOR, and NF‐κB pathways. This leads to a transition of disc cells from a matrix anabolic phenotype to a matrix catabolic and proinflammatory phenotype, ultimately leading to IVDD.

## Relationships between Mitochondria and ROS

### Mitochondria as the Main Site of Intracellular ROS Production

Mitochondria are the centers of oxidative metabolism, as 0.15% of oxygen consumption is converted to ROS.^36^ While this may seem like a small amount, 0.15% of total oxygen consumption represents a large amount of ROS. Mitochondria continuously produce ROS throughout the cell life cycle, which induces chronic age‐related oxidative stress, especially in mtDNA, that results in oxidative modification of bases or deletions.^37^

**Fig. 2:**
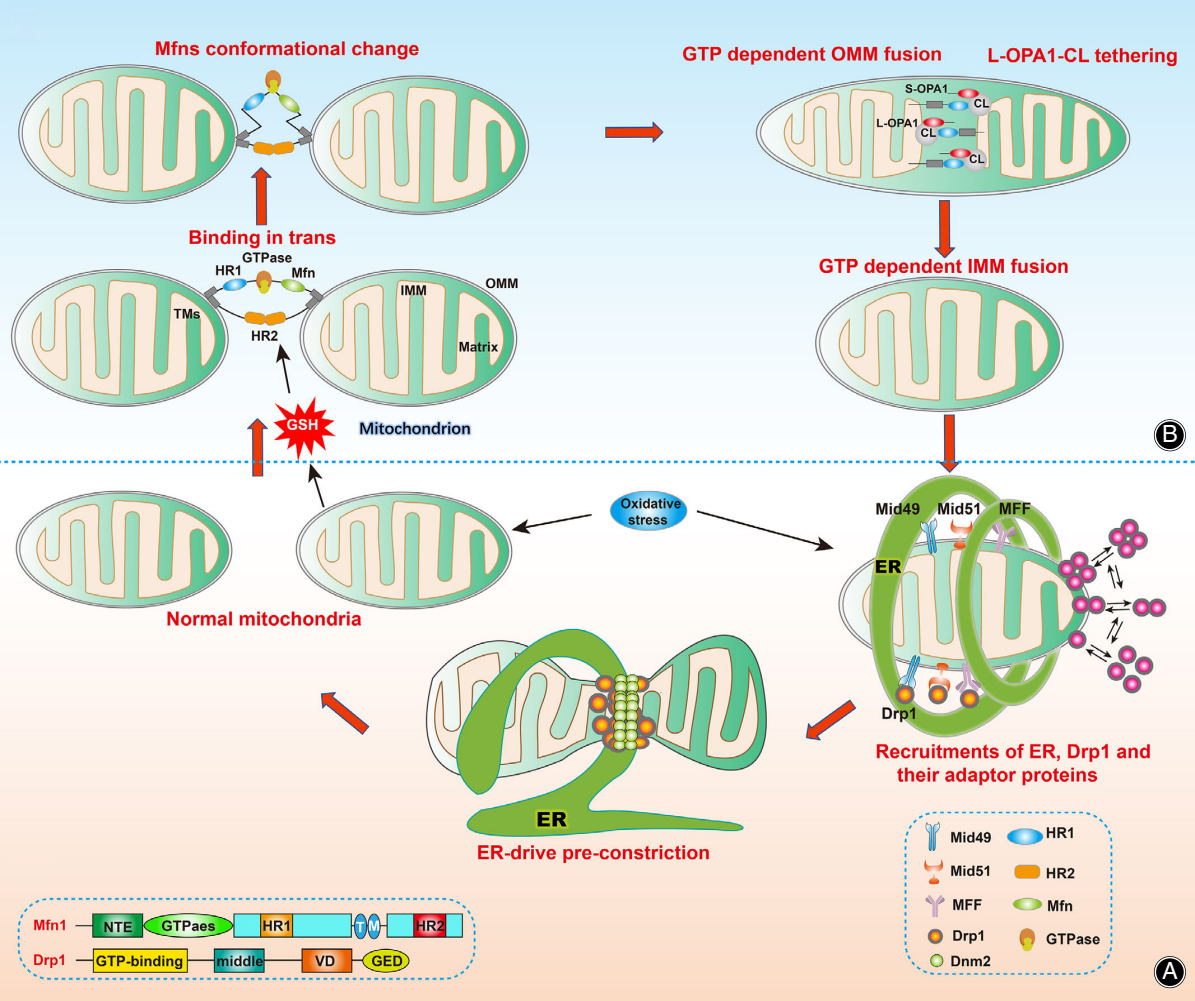
*Schematic diagram of ROS formation within the mitochondria*. The electron transport chain (ETC) and mitochondria‐localized proteins are involved in ROS production. The ETC is composed of five multi‐subunit enzyme complexes. Electrons transfer along the ETC coupled with proton transmission through the intima creates an electrochemical gradient between the intermembrane space and the matrix. Complex I, II and III produce O2‐ during electron transfer. Mitochondria‐localized proteins such as p66shc, NOX4, and MAOs are also involved in ROS production.

The transmembrane potentials of mitochondria control the production of ROS and are associated with the activity of AMP‐activated protein kinase (AMPK).^38^ The electron transport chain (ETC), an important ROS production site, is composed of five multi‐subunit enzyme complexes located in the inner mitochondrial membrane (IMM).^39^ The two most widely studied sites of the mitochondrial respiratory chain are complex I and III.^40^ For complex I, ROS is thought to originate from either reduced flavin mononucleotide or the N‐1a and N‐1b iron–sulfur clusters.^41^, ^42^ For complex III, ROS has been suggested to be produced at the ubiquinol oxidation site (ubiquinol: cytochrome c oxidoreductase).^43^ Complex II is believed to be associated with IVDD because the catalytic activity of complex II changes from succinate dehydrogenase to fumarate reductase under hypoxic conditions. This change is associated with increased ROS production.^44^ In addition to the complexes in the respiratory chain, many mitochondria‐localized proteins are also involved in ROS production, including p66shc, NOX4, and monoamine oxidases (MAOs). Among them, p66shc is a member of the Src homology 2 domain and collagen‐homology region (Shc) family that localizes to the mitochondrial intermembrane space and oxidizes cytochrome c to stimulate ROS production.^45^, ^46^ P66shc has been shown to participate in the regulation of mitochondrial homeostasis in NP cells and play an important role in IVDD.^47^ (Figure 2).

### Mitochondria as the Primary Target of Reactive Oxygen Species Attack

Mitochondria are also the main targets of ROS attack.^45^ Under physiological conditions, there exists a firm antioxidant defense in response to irritation of ROS in the human body. This protective mechanism is believed to be derived from the antioxidant system and the nuclear factor erythroid 2‐related factor (Nrf2)/heme oxygenase (HO‐1) signaling. The well‐established antioxidant system mainly includes antioxidant enzymes and small molecular weight molecules. Antioxidant enzymes represent the first line of defense against these toxic reactants and primarily include superoxide dismutase (SOD), glutathione peroxidase (GSH‐Px), methionine sulfoxide reductase (Msr), and peroxidases. SOD is ubiquitous within mitochondria of NP cells and is divided into two main isoforms: CuZnSOD and MnSOD. Msr is a repair enzyme that removes ROS by reducing methionine residues in oxidized proteins, which is down‐regulated in human senescent AF cells, making them more prone to oxidative damage.^48^ Nonenzymatic antioxidants, the second line of defense against free radicals, are also widespread in NP cells, predominantly in the cytoplasm. They act by rapidly reducing and inactivating free radicals and oxidants, mainly including vitamins C and E, β‐carotene, lipoic acid, ubiquinone, carotenoids, ascorbic acid, uric acid, and glutathione.^49^ In addition to the classical antioxidant system, an emerging role of the Keap1/Nrf2/HO‐1 signaling axis has been elucidated. Nrf2 is an inducible protein that normally binds to Keap1 in the cytosol. When disturbed by ROS, Nrf2 gets released from the sensor Keap1 and subsequently binds to antioxidant response elements (ARE) in the nucleus, thereby inducing the expression of detoxification genes that include phase II enzymes and antioxidant proteins, such as NAD(P)H: quinone oxidoreductase 1 (NQO1), glutamate‐cysteine ligase catalytic subunit (GCLC) and a modifier subunit (GCLM), and heme oxygenase‐1 (HO‐1). Particularly, HO‐1 is a redox‐sensitive inducible stress protein downstream of the Nrf2‐Keap1 axis that can curtail the cytotoxicity of various sources of oxidative stress and inflammation. To be specific, HO‐1 degrades heme into CO, iron, and biliverdin, which functions synergistically to constitute an antioxidant stress cellular defense mechanism for scavenging ROS, and detoxifying electrophiles and xenobiotics.^50^, ^51^, ^52^, ^53^, ^54^

The production of mitochondrial ROS is physiological and plays a vital role in many cellular functions. Moderate levels of mitochondrial ROS can activate the antioxidant compensation mechanism, protecting organelles from the harmful effects of ROS and ultimately achieving metabolic balance.^47^ However, when external conditions change, increased ROS production can induce mitochondrial membrane depolarization. Oxidative stress is induced when excessive ROS production outpaces antioxidant defenses. As the “first battlefield” of ROS, morphological and functional changes in mitochondria can directly affect the biological functions of NP and AF cells, resulting in an overproduction of MMPs and degradation of ECM.^55^ Therefore, the dysfunction of mitochondria plays an important role in oxidative stress‐mediated IVDD.

### Mitochondrial Dynamics

Mitochondria can change their shape, distribution, and size in the form of coordinated cycles of fission and fusion during many cellular processes such as cell cycle, apoptosis, and immune responses, referred to as mitochondrial dynamics. The dynamics consists of two major processes, namely mitochondrial fission and fusion. These dynamic shifts in balance not only ensure mitochondrial function but also respond to the needs of the cell by adapting to the availability of nutrients and the metabolic state of the cell.^56^ When the level of oxidative stress increases, it leads to an imbalance of mitochondrial fission and fusion, which is often linked with mitochondrial fragmentation, thus leading to mitochondrial dysfunction.^57^

#### Mitochondrial Fusion

Mitochondrial fusion, characterized by the division of one mitochondrion into two offspring mitochondria, occurs in three successive steps: (i) binding of the two mitochondria in trans; (ii) docking of the two membranes, which increases the contact surface area and reduces the distance between the two membranes; and (iii) eventually the fusion of the two OMMs due to conformational changes mediated by Mfn1 and Mfn2.^58^ Additionally, Mfn2 is a key regulator of mitochondrial–endoplasmic reticulum (ER) contact site connectivity.^59^ Mfn2 also functions as a scaffold protein for Parkin translocation after mitochondrial injury, which will be discussed later in the mitophagy section.^60^ Studies have demonstrated the role of ROS in promoting mitochondrial fusion.^61^, ^62^ In oxidized environments, two cysteines located in the C‐terminal region can be oxidized by increased levels of oxidized GSH, resulting in the binding of the outer membrane of two mitochondria in trans. Next, Mfn conformational changes induced by GTP binding and/or hydrolysis result in increased mitochondrial docking and membrane contact sites. Finally, GTP‐dependent oligomerization or GTPase‐dependent power stroke results in OMM fusion.^63^, ^64^

After the completion of OMM fusion, the IMM begins to fuse under the control of the large GTPase OPA1 and intima‐related lipids. OPA1 contains at least two proteolytic cleavage sites, S1 and S2 sites, which can generate shorter and soluble fragments under the control of two membrane‐bound metalloproteases, OMA1 and Yme1L.^65^, ^66^ Studies have found that in oxidative stress conditions, OMA1 plays a more important role than does Yme1L during IMM fusion progression.^67^ Afterwards, the large GTPase OPA1 is divided into at least five fragments, with the two highest molecular weight forms identified as L‐OPA1 and the other three as S‐OPA1. In addition, lipid components in the IMM, such as cardiolipin (CL), play a key role in membrane remodeling and dynamics. CL is necessary for the assembly and stabilization of large protein complexes such as mitochondrial contact sites and cristae tissue systems.^68^ Under oxidative stress conditions, a heterotypic interaction between L‐OPA1 and CL, which is promoted by S‐OPA1, drives IMM fusion.^69^ However, the spatial structure of OPA1 has not yet been identified, as the process of IMM fusion is currently based on the topology model. Thus, further studies are warranted.

#### Mitochondrial Fission

Mitochondrial fission is a multi‐step process, in which recruitment of the large GTPase Drp1 plays a crucial role. Drp1 exists in the cytoplasm and is dynamically recruited into the mitochondrial and peroxisome membranes to oligomerize and drive membrane contraction in a GTP‐dependent manner.^57^ During mitochondrial division, Drp1 is recruited to the OMM, where it forms a circular structure around the mitochondria and contracts after GTP hydrolysis, resulting in narrowing of the membrane^70^, ^71^ Drp1 is phosphorylated by cdk1/cyclin B kinase in a serine 616‐dependent manner.^72^ At the OMM, the middle domain induces conformational changes of Drp1 into Drp1‐oligomeric helices, after which the Drp1 oligomers move laterally along the mitochondrial tubule, induce constriction, and eventually fission.^73^

Because Drp1 lacks a domain for binding membrane phospholipids directly, its recruitment to OMM requires adaptor proteins. Recently, some studies have reported that the mitochondrial dynamics proteins 49 and 51 (MiD49 and MiD51) and mitochondrial fission factor (MFF) act as adaptor proteins for Drp1 recruitment and activity in mammals.^74^, ^75^ MFF is a substrate of the cellular energy sensor AMP‐activated protein kinase (AMPK) in the presence of mitochondrial dysfunction and decreased intracellular ATP/AMP ratio.^76^ MiD49 and MiD51 recruit Drp1 to OMM and then facilitate oligomerization. MFF selectively recruits oligomeric and active forms of Drp1.^77^ However, the precise mechanisms by which these adapters recruit Drp1 are not clear, and further experimental proof is needed.

Recruitment of Drp1 to the IMM leads to liposome tubulation but not scission, as final fission requires an additional process.^78^ Recent research has suggested that the canonical protein Dnm2, which is soluble in endocytic vesicles, is used to catalyze this last step.^79^ Dnm2, a GTP‐dependent enzyme, is assembled in a collar‐like structure around the constricting lipid “necks” of budding membrane‐bound vesicles.^58^ After Drp1 enters the IMM, Dnm2 is transiently and specifically recruited to the ER, after which Drp1 induces constriction sites, leading to fission.^79^

It is worth noting that ER plays an important role in mitochondrial fission. In fact, when Drp1 is recruited to the OMM, ER also approaches the mitochondrial periphery and is wrapped around mitochondria, causing mitochondrial constriction.^80^ This step reduces the mitochondrial diameter from about 300–500 nm to approximately 150 nm to allow the formation of Drp1‐oligomeric rings.^81^ These ER contact sites not only help shrink mitochondria but also serve as important metabolite and information exchange sites, promoting membrane remodeling and division. Besides serving as a recruitment site for Drp1 and its adaptor proteins MiD49 and MiD51, ER contact sites also exhibit phospholipid and Ca^2+^ transfer.^82^, ^83^ The actin‐nucleating proteins inverted‐formin 2 (INF2) and mitochondrial Spire1C regulate the actin assembly required for mitochondrial constriction at mitochondria–ER contact sites.^84^ Finally, Drp1 oligomerizes F‐actin in the mitochondria, leading to OMM rupture and mitochondrial fission.^85^

**Fig. 3:**
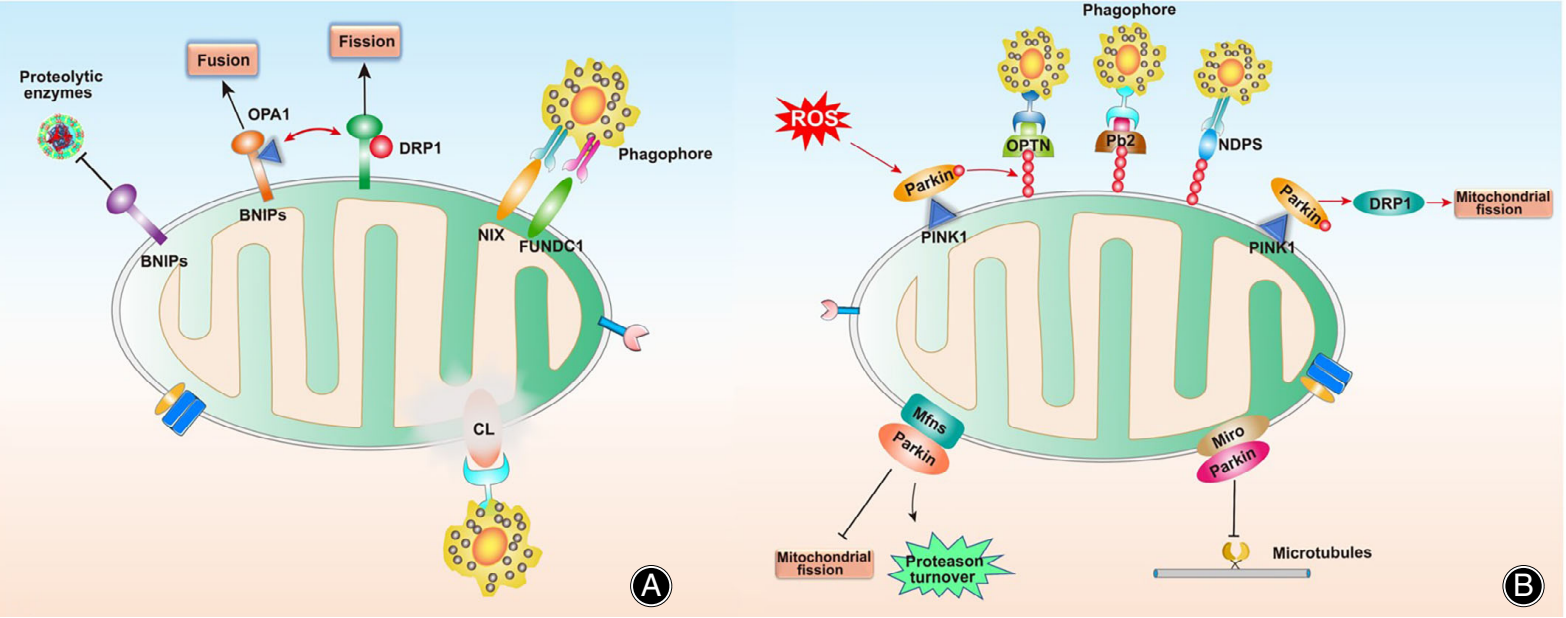
Schematic diagram of mitochondrial dynamics. (A) Domain structure of Mfn1 and Drp1. NTE, N‐terminal extension; HR1, heptad repeat 1; HR2, heptad repeat 2; TM, transmembrane domain; GED, GTPase effector domain; VD, variable domain. (B) In oxidized environments, two cysteines located in the C‐terminal region of Mfn can be oxidized by increased levels of oxidized GSH, resulting in the binding of the outer membrane of two mitochondria in trans. Mfn conformational changes induced by GTP binding and/or hydrolysis results in increased mitochondrial docking and membrane contact sites. GTP‐dependent oligomerization or GTPase‐dependent power stroke results in OMM fusion. The IMM's fuse is under the control of the large GTPase OPA1 and lipid components, like cardiolipin (CL). During mitochondrial division, Drp1 and ER are recruited to the OMM where they form a circular structure around the mitochondria with the help of MiD49, MiD51 and MFF, resulting in narrowing of the membrane at the mitochondria–ER contact sites in a Ca2 + −dependent process. After Drp1 enters the IMM, Dnm2 is transiently and specifically recruited to the ER, after which Drp1 induces constriction sites, leading to fission.

At present, while the role of Drp1 and ER in mitochondrial division is understood, Drp1 regulation in the mitochondria of NP cells has not been demonstrated. Moreover, current research is limited to the fission mechanism of OMM, with the IMM fission mechanism remaining unclear and needing further study. (Figure 3).

Overall, mitochondria are highly dynamic organelles that maintain their morphology, size, and distribution through fusion and fission under physiological and pathological conditions. Changes in mitochondrial morphology as well as abnormal mitochondrial morphology have been observed in NP cells within IVDs exposed to oxidative stress. It has been demonstrated that mitochondrial fusion and fission are out of balance during this pathological process, leading to abnormalities in mitochondrial shape, size, quantity, and most importantly, quality, which ultimately affect the energy metabolism of the cell.

#### Mitophagy

Mitophagy, a special form of autophagy, is the selective removal of dysfunctional or superfluous mitochondria to maintain mitochondrial quality and needs in cells, especially in persistent stimulation, like stress.^86^ Impaired mitophagy leads to the gradual accumulation of dysfunctional mitochondria, which induces NP cell apoptosis and ECM degradation, eventually leading to disc degeneration.^87^, ^88^ Mitophagy can be roughly divided into three categories: (i) putative kinase 1 (PINK1)–Parkin‐mediated; (ii) Parkin‐independent; and (iii) protein‐mediated mitophagy.

#### PINK1–Parkin‐dependent Mitophagy

PINK1–Parkin‐dependent mitophagy is ubiquitin (Ub)‐dependent and occurs *via* phosphatase and tensin homologue (PTEN)‐induced PINK1–Parkin regulation.^57^ In functional mitochondria, PINK1 is transported to the IMM and cleaved by several proteases in a membrane potential‐dependent manner.^89^ After being hydrolyzed, PINK1 is degraded by the Ub‐proteasome system to maintain PINK1 at low levels.^90^ However, if the mitochondrial membrane potential is decreased, PINK1 cannot enter the IMM, resulting in an accumulation of PINK1 at the OMM.^91^ PINK1 is then activated by auto‐phosphorylation on the OMM, attracting Parkin translocation to the OMM^92^ and triggering its Ub E3 ligase activity.^93^ PINK1 has been shown to activate Ub and poly‐Ub chains of dysfunctional mitochondrial proteins, and ubiquitinated Ub can further activate Parkin; this activation is considered a positive feedback loop that amplifies mitophagy signals.^94^ Ubiquitinated Ub and poly‐Ub chains may act as signals of damaged mitochondria by binding to light chain 3 (LC3) and providing recognition sites for autophagy adaptors, such as sequestosome 1 (SQSTM1)/p62, optineurin (OPTN), and calcium binding and coiled‐coli domain 2/nuclear dot protein 52 (CALCOCO2/NDP52).^95^, ^96^

This form of mitophagy is mainly associated with mitochondrial quality control mechanisms, such as those affecting the production of mitochondria‐derived vesicles and mitochondrial dynamics.^97^ In fact, oxidative stress causes dysfunctional mitochondria, and their mitophagy mainly occurs through PINK1–Parkin. PINK1 can indirectly promote the activity of Drp1, thereby promoting mitochondrial fission and facilitating mitophagy.^98^ In addition, Parkin‐dependent proteasomal turnover of Mfns can interrupt the fusion of damaged mitochondria and isolate damaged mitochondria from the healthy mitochondrial pool.^99^ PINK1–Parkin‐mediated Mfn2 and other OMM‐specific protein degradation separates damaged mitochondria from a healthy mitochondrial network.^100^ It has been demonstrated that Mfn2 overexpression promotes ROS‐dependent mitophagy *via* the PINK1–Parkin pathway in human NP cells.^101^

Therefore, PINK1–Parkin‐dependent mitophagy not only marks recognition sites for autophagosomes through ubiquitinated Ub and poly‐Ub chains on OMM but also promotes mitochondrial fission, inhibits fusion, and inhibits the contact site formation between mitochondria, ER, and the cytoskeleton. As a result, the mitochondrial structure become smaller and more immobile, making them easier for autophagosomes to swallow. Nevertheless, the substrate of Parkin on the OMM and its degradation mechanism remain unknown, and thus regulating Parkin activity on the OMM is not yet possible. These questions require further study.

#### Parkin‐independent Mitophagy

In PINK1–Parkin‐dependent mitophagy, the ubiquitin E3 ligase of Parkin plays a crucial role. Several other ubiquitin E3 ligases, such as Gp78, SMURF1, SIAH1, MUL1, and ARIH1, also function in mitophagy regulation.^102^, ^103^, ^104^ Once these ubiquitin E3 ligases are activated on the OMM, they can also ubiquitinate Ub and poly‐Ub chains to mark damaged mitochondria, provide recognition sites for autophagosomes, and help recruit autophagy adaptors. The autophagy adaptor directly interacts with autophagosome light chain 3 (LC3) through its LC3 interaction region (LIR) motif, anchoring Ub‐labeled mitochondria into the autophagosomes. However, the current understanding of this complex is insufficient; for example, what is the initial signal of TBK1 activation and does TBK1 undergo phagocytosis? These questions require further research.

#### Ubiquitin‐independent Mitophagy

**Fig. 4:**
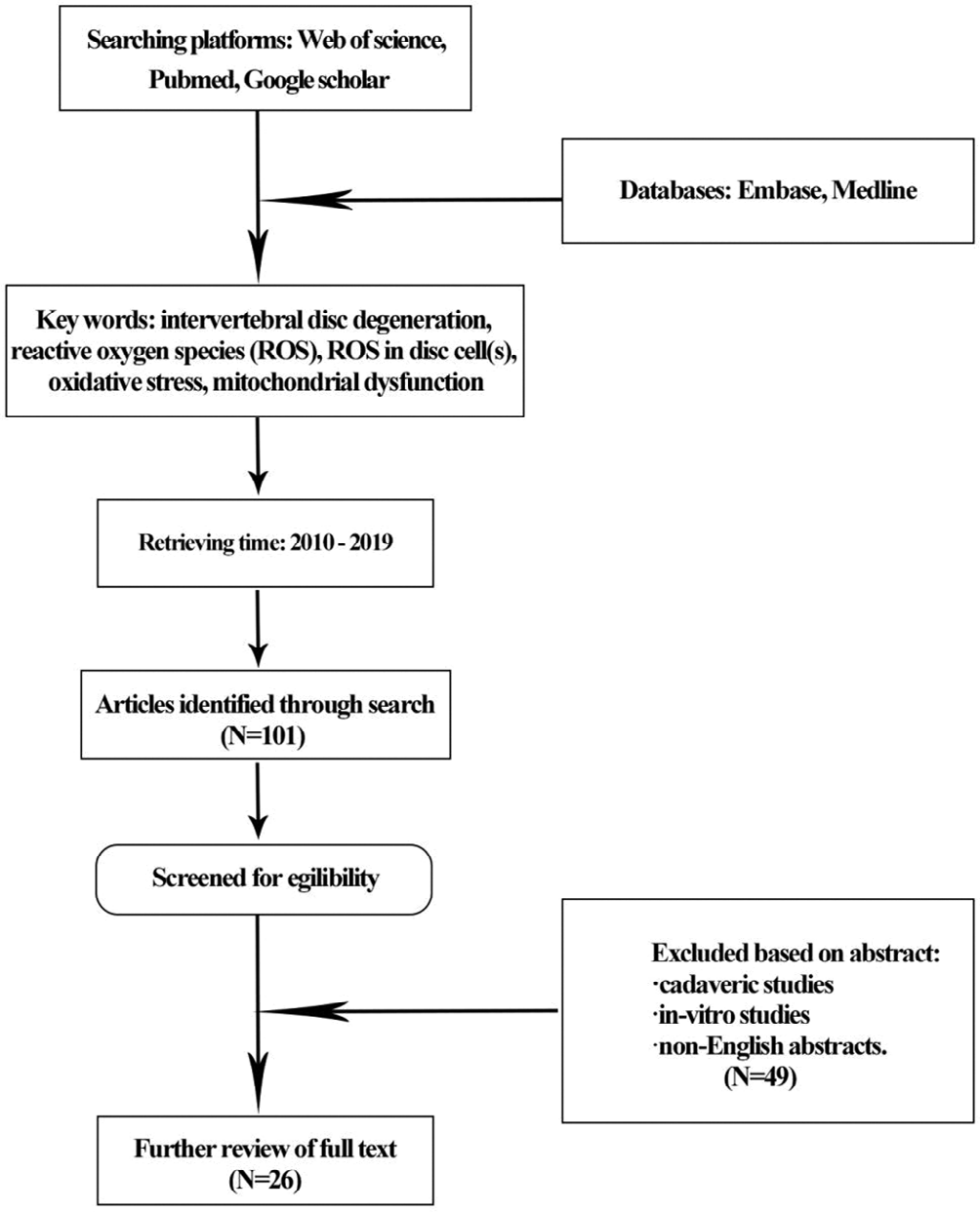
Illustration of the correlation between mitochondrial dynamics and mitophagy. (A) Mitochondrial fusion comprises of the fusion of the two OMMs (mediated by Mfn1 and Mfn2), and IMM fusion (mediated by OPA1). Mitochondrial fission includes the recruitment of the Drp1 which exists in the cytoplasm and is recruited into the mitochondrial membranes to oligomerize and drive membrane contraction. (B) In the process of mitophagy, ubiquitinated Ub and poly‐Ub chains act as signals for damaged mitochondria by providing recognition sites for autophagy adaptors (p62, OPTN, NDP, and LC3). PINK1 can promote the activity of Drp1, thereby promoting mitochondrial fission and facilitating mitophagy. Parkin‐dependent proteasomal turnover of Mfns can interrupt the fusion of damaged mitochondria and isolate damaged mitochondria from the healthy mitochondrial pool. PINK1–Parkin‐mediated Mfn2 and other OMM‐specific protein degradation, which separates damaged mitochondria from a healthy mitochondrial network.

The two previously mentioned modes of mitophagy require ubiquitination of Ub and poly‐Ub chains on the OMM, however, some mitochondrial proteins can serve as autophagy receptors, targeting dysfunctional mitochondria directly to autophagosomes for degradation.^105^ These LIR‐containing proteins can be directly linked to LC3 and GABARAP autophagosomal membrane proteins, mediating mitophagy.^106^ These proteins include BCL2‐interacting protein 3 (BNIP3), NIP3‐like protein X (NIX), DCT‐1, and FUN14 domain‐containing protein 1 (FUNDC1). Specifically, BNIP3 maintains PINK1 stability and aggregation mainly by inhibiting the activity of proteolytic enzymes, after which mitophagy occurs through the PINK1–Parkin‐dependent pathway.^107^ Meanwhile, by regulating the decomposition and release of OPA1, BNIP3 can promote the recruitment of Drp1 to the mitochondrial surface, promoting mitochondrial fission and fusion.^108^ In the case of oxidative stress, damaged mitochondria show a decrease in mitochondrial membrane potential, and NIX directly combines with LC3 through the LIR motif.^109^ Although the signaling molecules involved in this process are not well understood, studies have shown that NIX is associated with mitochondrial localization and removal.^110^ (Figure 4).

#### Mitophagy in Oxidative Stress‐mediated IVDD

Mitophagy is constantly occurring in cells and completes the quality control processes of the mitochondrial system that ensure cell energy metabolism and tissue homeostasis by the timely removal of dysfunctional mitochondria. Mitochondrial injury induced by oxidative stress is more acute than that induced by programmed mitochondrial death. In IVDD, tumor necrosis factor (TNF) is a generally elevated cytokine and induces the significant up‐regulation of PINK1 in NP cells.^87^ Moreover, the degree of PINK1 up‐regulation is positively correlated with IVDD processes. Oxidative stress induces excessive mitophagy and further leads to NP cell death.^111^ Subsequent studies have found that oxidative stress induces changes in mitochondrial dynamics and thus increases mitophagy. If PINK1 activity is inhibited, mitophagy is reduced, resulting in increased NP cell senescence.^112^ This suggests that PINK1–Parkin‐dependent mitophagy has a protective effect on the mitochondrial system through timely clearance of damaged mitochondria in the context of oxidative stress. The regulatory relationships between PINK1 and Mnf2 have been described above. Moreover, when intracellular ROS increases in NP cells, Mnf2 expression also increases, promoting PINK1–Parkin‐dependent mitophagy.^101^

It was clear that mitophagy can remove damaged mitochondria and maintain homeostasis of the mitochondrial system. However, excessive mitophagy leads to excessive mitochondrial clearance, accelerated senescence, and eventually NP and AF cell death.^113^ Although the role of mitophagy in IVDD has been studied, most studies were performed *in vitro* or in rats. In addition, how mitophagy changes its role and what factors are regulated in different IVD lesions requires further elucidation.

## IVDD Therapeutic Strategies Addressing Mitochondrial Dysfunction

During oxidative stress‐induced IVDD, mitochondrial dysfunction has a critical impact on the function of NP and AF cells and plays an important role in IVDD. In this section, we will discuss several mitochondrial function‐related therapies that have great potential as IVDD treatment.

### Mitochondria‐targeted Antioxidants

Mitochondria‐targeted antioxidants can specifically enrich their concentrations in mitochondria, playing an efficacious antioxidant role. Considering the important roles of mitochondria during oxidative stress, such antioxidants have therapeutic significance for the treatment of oxidative stress‐induced IVDD. For example, mitoquinone (MitoQ) is a mitochondria‐targeted antioxidant composed of coQ10 and a triphenylphosphine (TPP) cation that easily accumulates in the mitochondria, making it more effective than non‐targeted antioxidants in preventing mitochondrial oxidative damage.^114^ MitoQ has been shown to attenuate mitochondrial dysfunction and human NP cell apoptosis.^115^ A recent study showed that MitoQ restores mitochondrial dynamic balance, alleviates impairment of mitophagosome‐lysosome fusion and lysosomal function damage, and enhances Nrf2 activity. MitoQ can promote clearance of dysfunctional mitochondria caused by oxidative stress and increase the survival time of NP cells in a ROS‐rich microenvironment; these results are consistent with those of *in vivo* experiments.^115^ However, there is limited research on such antioxidants, warranting further research.

### Non‐Mitochondria‐targeted Antioxidants

#### The Sirtuin Family Pathway Regulation

Sirtuins are NAD + ‐dependent histone deacetylases—homologous to the yeast silent information regulator 2 (Sir2)—that are associated with the cell life cycle. Sirtuin has been shown to play an important role in a variety of oxidative stress‐induced degenerative diseases, including IVDD.^116^ Of the sirtuin family, SIRT1, SIRT2, SIRT3, and SIRT6 regulate senescence in NP and AF cells by participating in inflammation, oxidative stress, and mitochondrial dysfunction.^117^, ^118^, ^119^, ^120^

SIRT1 inhibits the NF‐κB signaling pathway by inactivating p53; inhibits the peroxisome proliferator‐activated receptor gamma coactivator 1‐alpha (PGC‐1α) signaling pathway by inducing deacetylation of PGC‐1α; and increases mitophagy to enhance NP cell resistance to oxidative stress.^121^, ^122^ In addition, SIRT1 has been shown to reduce oxidative stress‐induced senescence in human CEP cells through the p53/P21 pathway.^35^ SIRT1 can also directly affect mitophagy in NP cells.^123^ SIRT2 is up‐regulated by oxidative stress and protects AF cells from oxidative stress‐induced apoptosis by inhibiting mitophagy through PGC‐1 regulation. SIRT2 can also delay NP cell senescence by inhibiting the p53/P21 pathway.^124^ SIRT3, which maintains intracellular ROS homeostasis by regulating mitochondrial function in NP and AF cells,^125^ has strong deacetylase activity and is directly regulated by the ratio of NAD+/NADH.^126^ If mitochondria produce too much ROS, SIRT3 expression is increased, which up‐regulates the expression of FOXO3a‐dependent genes (e.g., SOD2 and catalase).^127^ Meanwhile, SIRT6 expression is normally low in senescent human NP cells. When SIRT6 is overexpressed, NP cell apoptosis and stress‐induced senescence can be prevented.^128^ However, the specific role of SIRT6 in IVDD and its regulatory pathways are not fully understood, warranting further research.

Resveratrol (3,4′,5‐trihydroxystilbene) is a natural polyphenolic compound and a strong activator of SIRT1.^129^ Resveratrol can increase SIRT1 expression and enhance the synthesis of NP cell ECM through the Wnt/β‐catenin signaling pathway.^130^ This is consistent with an *in vivo* experiment, where treating degenerative NP cells with resveratrol increases extracellular proteoglycan and type II collagen synthesis.^131^ Thus, resveratrol has therapeutic significance for IVDD. Melatonin (N‐acetyl‐5‐methoxytryptamine) is an endocrine hormone and activator of SIRT1. Studies showed that melatonin can induce the PRKN‐dependent activation of mitophagy and reduce the release of ROS and apoptosis factors, thus inhibiting oxidative stress‐induced NP cell apoptosis and ECM degradation, whereas the mitophagy inhibitor (CsA) abolishes these beneficial effects. In line with this, *in vivo* studies have demonstrated that melatonin may have a protective effect on IVDD in puncture‐induced rat models.^132^, ^133^, ^134^ 1,4‐Dihydropyridine (DHP) is a SIRT1 activator that significantly increases the levels of SIRT1 and the antioxidant protein SOD1 and significantly protects against ROS‐induced degradation of collagen II and aggrecan.^135^ Moreover, it has been confirmed *in vivo* that DHP inhibits IL‐1β‐induced ROS accumulation and ECM degradation by activating SIRT1 in human NP cells.

Nicotinamide mononucleotide (NMN), a SIRT3 activator, can enhance the biological effects of NAD^+^. NMN can rescue human NP cell apoptosis through the AMPK‐PGC‐1α pathway under an oxidative stress microenvironment.^136^ Honokiol (C_18_H_18_O_2_) is a natural small‐molecule compound extracted from the roots and bark of *Magnoliaceae* plants that has been shown to inhibit IVDD by activating SIRT3 through the AMPK‐PGC‐1α signaling pathway.^137^ Honokiol can enhance the antioxidant capacity of mitochondria in NP cells, promote mitochondrial fission and fusion, and prevent NP cells from undergoing apoptosis and senescence under oxidative stress. Moreover, Honokiol was reported to upregulate the expression of mitophagic markers, BNIP3 and BNIP3L, while SIRT3 knockdown diminished this Honokiol‐mediated mitophagy in NP cells.^138^

Induced pluripotent stem cell‐derived small extracellular vesicles (iMSC‐sEVs) are novel therapeutic strategy for IVDD treatment. A recent study has disclosed the molecular mechanism of iMSC‐sEVs in a rat puncture model. To be specific, iMSC‐sEVs can restore the NP cell senescence by delivering the microRNA‐105‐5p to senescent NP cells, which results in the SIRT6 pathway activation *in vitro*.^139^

Collectively, it has been confirmed that SIRT1, 2, 3, and 6 are involved in IVDD during oxidative stress, highlighting them as specific and effective targets for IVDD treatment. However, their specific molecular mechanisms require further study. In addition, whether SIRT4 and SIRT5 and their related activators have regulatory effects on IVDD should be further clarified.

#### The Nrf2 Pathway Regulation

Icariin is a prenylated flavanol glycoside isolated from *Epimedium* plants and a traditional Chinese medicine. Icariin has been shown to have antioxidant effects on human NP cells through its regulation of inducible nitric oxide synthase (iNOS), nitric oxide (NO), and catabolic enzyme production.^140^ Icariin was also found to up‐regulate SIRT6 expression in murine heart tissues.^141^ Moreover, icariin was demonstrated to exert protective effects on ROS‐induced oxidative injury and mitochondria‐mediated apoptosis in human NP cells through the Nrf2 signaling pathway.^142^ However, further studies are needed to elucidate the mechanisms and targets of icariin in detail.

Kinsenoside (Kin), extracted from the plant *Anoectochilus roxburghii*, is a medicinal herb widely distributed in the tropics and is known as the “medicine of kings” because of its extensive pharmacological action.^143^ Kin functions include antioxidant, anti‐inflammatory, and anti‐apoptotic effects, among others.^144^ Studies have confirmed that Kin can activate the AKT‐ERK‐Nrf2 signaling pathway in human NP cells, reducing mitochondrial dysfunction, apoptosis, and senescence *in vitro*.^145^ However, the therapeutic efficacy of Kin has only been confirmed in the rat model, and thus its therapeutic efficacy in human tissues requires further verification.

Lycopene, a naturally occurring carotenoid that can be extracted from tomatoes, tomato products, and other red fruits and vegetables, possesses the strongest antioxidant capacity of natural carotenoids.^146^, ^147^ Lycopene was found to effectively attenuate H_2_O_2_‐induced human NP cell apoptosis by activating Nrf2, which is closely associated with p62; increased p62 expression can inhibit autophagy.^148^ Moreover, lycopene was able to prevent the ROS‐induced degradation of the ECM of NP cells.^149^ However, this study was carried out *in vitro* and requires further confirmation *in vivo*.

Cardamonin (CAR), a chalcone extract from Alpinia katsumadai and other plants, exhibits protective effects on NP cells *in vitro* and in a puncture‐induced rat IVDD model *in vivo*. The treatment of CAR significantly counteracts the ECM degradation and the release of inflammatory cytokines by activating the Nrf2/HO‐1 signaling axis in rat NP cells. Further, the intragastric administration of CAR in a rat model demonstrates its significant potential for IVDD therapy.^150^

#### Other Pathways Related to Mitochondrial Regulation

Berberine, an isoquinoline alkaloid isolated from *Coptidis 19hizome* and *Cortex phellodendri*,^151^ has a wide range of pharmacological activities including anti‐inflammation, antioxidation, and hypoglycemia by modulating oxidative stress.^152^ Berberine has been shown to regulate ROS‐induced NP cell apoptosis by modulating ER stress, thereby affecting mitochondrial dynamics and autophagy; the important role of the ER in mitochondrial fission has been described.^153^

Urolithin A, a metabolite of ellagitannins and ellagic acid, exerts protective impacts against mitochondrial dysfunction by promoting mitophagy *in vivo* and *in vitro*. Specially, the AMPK signaling initiates the urolithin A‐mediated mitophagy.^154^

Mangiferin is a potent natural compound isolated from the Mangifera indica plant that can protect against multiple diseases through combating oxidative stress and mitochondrial dysfunction. Recently, its protective effect on IVDD was verified in a rat needle puncture model. Together with the *in vitro* evidence, mangiferin can antagonize mitochondrial ROS in NP cells and reverse the loss of major intervertebral disc components through inhibiting the NF‐κB signaling pathway.^155^

Quercetin, a naturally occurring flavonoid, has been extensively explored in degenerative diseases due to its antioxidant ability. It is reported to partially inhibit the p38‐MAPK pathway in NP cells, which significantly alleviates oxidative stress and prevents ECM degeneration in a rat tail puncture induced model of IVDD.^156^ (Table 1).

**TABLE 1:** IVDD therapeutic strategies addressing mitochondrial dysfunction

Type	Target	Name	Therapeutic sites	Main effects	References
Mitochondria‐targeted antioxidants	Mitochondria	MitoQ	NP, AF and CEP cell	↑ mitochondrial dynamic balance	^114^, ^115^
Non‐mitochondria‐targeted antioxidants	The sirtuin family	Resveratrol	NP cell	↑extracellular proteoglycan and type II collagen synthesis	^129^, ^130^, ^131^
		Melatonin	NP cell	↑ mitophagy ↓ ROS release and apoptosis factors	^132^, ^133^, ^134^
		DHP	NP cell	↓ ROS accumulation ↓ ECM degradation	^135^
		NMN	NP cell	↓ cell apoptosis	^136^
		Honokiol	NP cell	↑ mitophagy ↑ mitochondrial fission and fusion	^137^, ^138^
		iMSC‐sEVs	NP cell	↓ cell senescence	^139^
	The Nrf2 pathway	Icariin	NP cell	↓ iNOS, NO, and catabolic enzyme production ↓ cell apoptosis	^140^, ^141^, ^142^
		Kinsenoside	NP cell	↓ cell apoptosis ↓ cell senescence	^143^, ^144^, ^145^
		Lycopene	NP cell	↑ p62 expression ↓ ECM degradation	^146^, ^147^, ^148^, ^149^
		CAR	NP cell	↓ ECM degradation ↓ inflammatory cytokines release	^150^
	ER stress	Berberine	NP cell	↑ mitochondrial fission ↓ cell apoptosis	^151^, ^152^, ^153^
Non‐mitochondria‐targeted antioxidants	The AMPK pathway	Urolithin A	NP cell	↑ mitophagy	^154^
	The NF‐κB pathway	Mangiferin	NP cell	↓ mitochondrial ROS	^155^
	The p38‐MAPK pathway	Quercetin	NP cell	↓ ECM degradation	^156^

Abbreviations: AF, annulus fibrosus; CAR, Cardamonin; CEP, cartilage endplate; ECM, extracellular matrix; ROS, reactive oxygen species; MitoQ, mitoquinone; SOD, superoxide dismutase; DHP, 1,4‐Dihydropyridine; NMN, nicotinamide mononucleotide; NP, nucleus pulposus; ER, endoplasmic reticulum.

## Summary and Future Perspectives

As a byproduct of aerobic respiration, ROS is constantly being produced and accumulated in the mitochondria, which is also the main target of ROS attack in cells. Under normal physiological conditions, ROS can be cleared timely through the antioxidant system that consists of antioxidant enzymes, nonenzymatic antioxidants, and the intracellular Nrf2/HO‐1 signaling pathway. However, with an increase in age, changes in disc stress, and other common pathogenic factors of IVDD, the intracellular ROS balance is disrupted, leading to mitochondrial dysfunction. Mitochondria can alter their own dynamics through fusion and fission, so that disabled mitochondria can be separated from the mitochondrial pool. Moreover, mitophagy, a specific type of autophagy, participates by clearing these dysfunctional mitochondria. Abnormalities in any of these processes affects the balance between ROS clearance and production, leading to a vicious cycle that results in the apoptosis of disc cells and degradation of the ECM. The excessive production and accumulation of intracellular ROS leads to IVDD mainly through the following signaling pathways: NF‐κB, MAPK, PI3K/Akt, phospholipase, and protein kinase C pathways.^157^

Currently, an increasing number of therapeutic strategies targeting oxidative stress has been brought up. Given the pivotal role of mitochondria in oxidative stress, therapies that address mitochondrial dysfunction may have substantial potential in IVDD therapeutics. Many studies have described the protective effects of mitochondria‐targeted and non‐targeted antioxidants on disc cells and animal IVDD models, however, there is a paucity of evidence on the treatment efficacy of these strategies in human patients, further clinical trials are required to demonstrate whether mitochondrial regulation can alleviate disease progression. Concerning the anatomical traits of intervertebral discs, efficient drug delivery systems also guarantee future research. Overall, multiple signaling pathways related to cell death and inflammation converge on mitochondria, and mitochondria play a pleiotropic part in disc cells, thus modifying mitochondrial dysfunction can be the focus of future IVDD research.

## Conflict of Interest

The authors confirm that there are no conflicts of interest.
